# Do prospective longitudinal studies of bipolar disorder support the hypothesis of neuroprogression?

**DOI:** 10.1192/j.eurpsy.2023.468

**Published:** 2023-07-19

**Authors:** I. Melle, T. V. Lagerberg, B. Etain, S. H. Lyngstad, K. F. Wold

**Affiliations:** ^1^Research and innovation, Oslo university hospital, Oslo, Norway; ^2^Centre Expert Trouble Bipolaire, Hôpital Lariboisière - F. Widal, Paris, France; ^3^Nydalen DPS, Oslo university hospital; ^4^Institute of clinical medicine, University of Oslo, Oslo, Norway

## Abstract

**Introduction:**

Bipolar I disorder is a mental disorder with the risk of severe clinical outcomes. Bipolar disorder was initially defined based on having a better outcome than schizophrenia. However, while recent longer-term findings in schizophrenia do not support neuroprogression, bipolar disorder is increasingly depicted as having neuroprogressive elements. There are, however, remarkably few prospective longitudinal studies of representative bipolar I cohorts followed from the first treatment.

**Objectives:**

To study the clinical development of a representative cohort of bipolar disorder patients recruited at their first treatment.

**Methods:**

Patients with DSM-IV Bipolar I or Bipolar NOS were consecutively recruited from in-and outpatient units in the larger Oslo area during their first treatment year and extensively clinically characterized at baseline. They then participated in personal one- and ten-year follow-ups.

**Results:**

Sixty-nine patients participated in the 10-year follow-up. Age at follow-up was 39.0 (+ 9.6) years, 59% were females. A total of 12% had unipolar mania, 58% had psychotic bipolar disorder, and 20% had experienced rapid cycling. At follow-up, 75% were in full affective remission, 60% had regained full functioning, and 54% were in stable full recovery.

Mood episode relapses clustered around the first episode. Despite occasional relapses, 2/3 were mainly euthymic during the follow-up period. A small sub-group was highly affected from the first 2-3 years of treatment, but there were no apparent signs of kindling effects or indications of neuroprogression

**Conclusions:**

The follow-up of this cohort of first-treatment Bipolar I patients does not support the hypothesis of neuroprogression.

**Disclosure of Interest:**

None Declared

